# HIV-related stigma experiences and coping strategies among pregnant women in rural Uganda: A qualitative descriptive study

**DOI:** 10.1371/journal.pone.0272931

**Published:** 2022-10-07

**Authors:** Judith Jolle, Amir Kabunga, Tonny Owili Okello, Esther Oloi Kadito, Jimmy Aloka, Geoffrey Otiti, Agnes Adong Aluku, Edward Kumakech, Samson Udho

**Affiliations:** 1 Department of Public Health, Faculty of Health Sciences, Lira University, Lira, Uganda; 2 Department of Community Psychology & Psychotherapy, Faculty of Health Sciences, Lira University, Lira, Uganda; 3 Department of Nursing & Midwifery, Faculty of Health Sciences, Lira University, Lira, Uganda; PLOS: Public Library of Science, UNITED KINGDOM

## Abstract

**Background:**

HIV-related stigma is a global problem among HIV clients with far-reaching effects including increased rates of mother-to-child transmission of HIV. However, HIV-related stigma experiences and coping strategies have received little attention, especially among pregnant women in rural settings. We explored the HIV-related stigma experiences and coping strategies among pregnant women in rural northern Uganda.

**Methods:**

This was a qualitative descriptive study conducted among HIV-positive pregnant women seeking care at Aboke Health Center IV, Kole district, northern Uganda. We conducted 12 in-depth interviews using a semi-structured interview guide. Data were analyzed using the inductive thematic approach of Braun and Clarke.

**Results:**

The age range of the 12 participants was 17 to 35 years while the average duration with HIV since diagnosis was five years. The majority of the participants were subsistence farmers who had attained a primary level of education. Social rejection and public ridicule were identified as HIV-related stigma experiences while ignoring, social support, and prayers were identified as HIV-related coping strategies among the study participants.

**Conclusion:**

Enacted HIV-related stigma is common among pregnant women in rural northern Uganda. Healthcare providers should work closely with HIV-positive women and other stakeholders to identify and strengthen HIV-related stigma coping strategies among pregnant women in rural settings.

## Introduction

HIV-related stigma remains a global public health problem with far-reaching consequences [1, 2]. HIV-related stigma refers to prejudice, negative attitudes, and abuse directed at people living with HIV and AIDS that is believed to be socially unacceptable [1, 2]. The stigma can either be a) enacted/experienced stigma- actual experiences of discrimination, b) anticipated/perceived/felt *stigma-*the fear of experiencing discrimination when associating with an HIV-positive person or testing positive, c) self/internalized stigma-acceptance of shame, blame, guilt and fear associated with being HIV-positive, and d) intersecting/layered stigma*-*HIV stigma that is layered on top of the pre-existing stigma that is frequently directed toward most-at-risk groups [1, 3].

Globally, one in eight people living with HIV experience some form of stigma [1]. The burden of stigma and discrimination is highest in Sub-Saharan Africa where the burden of HIV is high [4]. In Uganda, it is estimated that 33% of persons living with HIV have ever experienced some form of stigma [5]. The perpetual HIV-related stigma is partly because of the myths and misconceptions surrounding the transmission and prognosis of HIV/AIDS [5].

The stigma that accompanies being HIV positive has numerous negative ramifications such as loss of income and livelihood, loss of a marriage, and childbearing options, poor care within the health sector, withdrawal of caregiving in the home, loss of hope, feelings of worthlessness, and loss of reputation [6]. HIV-related stigma among pregnant women has also been associated with depression, violence, loss to follow-up, poor adherence to treatment, increased viral load, and increased risk of mother-to-child transmission of HIV [7, 8]. Ninety percent of pediatrics HIV infection is due to mother-to-child transmission which occurs during pregnancy, delivery, and breastfeeding [9]. Despite these challenges, limited studies have explored how pregnant mothers in rural settings living with HIV live and cope with these challenges.

The fight against HIV-related stigma has taken different strategies such as open campaigns against stigma and discrimination, legislation against stigma and discrimination, integration of services, and creation of communities of practices with an emphasis on experience sharing [9–12]. However, there remain gaps in addressing HIV-related stigma among pregnant women which may hamper the uptake of elimination of mother-to-child transmission (EMTCT) services thus increasing the rates of pediatric HIV. To address these gaps, there is a need to explore the HIV-related stigma experiences that pregnant mothers go through and how they cope with these experiences.

HIV-related stigma against people living with HIV (PLWHIV) have been extensively documented in Ethiopia, the United States of America, Zambia, South Africa, and Uganda [13–24]. However, most of these studies were quantitative, focused on the general HIV population, and gave little attention to how the victims cope with HIV-related stigma. In Uganda, there is a dearth of literature on HIV-related stigma among pregnant women since the country’s renewed interest in EMTCT. To achieve EMTCT, it is important to explore the HIV-related stigma experiences and coping strategies of pregnant women across cultural contexts within the country. The purpose of the current study was to explore HIV-related stigma experiences and coping strategies among pregnant women in Kole District, northern Uganda.

## Materials and methods

### Study design

A qualitative descriptive study [25, 26] was conducted in August 2020 to explore the HIV-related experiences and coping strategies of pregnant women in Kole District, rural northern Uganda. We used a semi-structured interview guide with open-ended questions to explore the phenomenon in its natural setting using face-to-face interviews. The interview guide was pilot tested among pregnant women who were not included in the study and revisions were made accordingly.

### Study setting

The study was conducted in the EMTCT clinic of Aboke Health Center IV which is located in Kole District, northern Uganda. Kole District is bordered by Lira District to the east, Apac District to the south, and Oyam District to the west and north. The district is located approximately 290 kilometers by road to Kampala, the capital city of Uganda. The EMTCT clinic offers antiretroviral therapy services to pregnant, lactating mothers, and HIV-exposed infants. The unit is run by midwives, counselors, and medical officers, and it operates on Wednesday and Friday from 9:00 to 17:00 hours.

### Study participants and sample size

The study was conducted among HIV-positive pregnant women who have ever sought EMTCT services at Aboke Health Center IV. The study included pregnant women who were 15 years of age and above and could speak either *Lango* or the English language. Pregnant women who were ill or busy at the time of data collection and did not provide consent to participate in the study were excluded. Purposive sampling was used to sample 15 participants who could speak to the research aims and have knowledge and experience of the phenomenon under scrutiny [27]. The principle of information power which indicates that the more information the sample holds, relevant for the actual study, the lower amount of participants is needed was used to estimate the sample size [28].

### Participants’ recruitment and data collection tool

The facility-community linkage facilitators helped the data clerks to recruit the eligible study participants who could express themselves in a manner that would allow us to capture a rich perspective of HIV-related stigma experiences and coping strategies of pregnant women. Data were collected using a semi-structured interview guide. The interview guide was developed by SU and AK and questions were adapted from the conceptual framework on HIV-related stigma and discrimination developed by Turan *et al*. in 2012 [29]. The interview guide had three sections comprising socio-demographic characteristics (age, level of education, marital status, number of children, occupation, and duration with HIV), HIV-related stigma experiences, and coping strategies. The socio-demographic characteristics had closed-ended questions while the questions on HIV-related stigma and coping strategies had open-ended questions.

### Data collection method and procedure

Data were collected using face-to-face in-depth interviews. The interviews were conducted in *Lango* and English language in a private setting within the health facility premises. To elicit information on HIV-related stigma experiences and coping strategies, the study participants were asked to describe their experiences as pregnant women who are HIV positive (S1 Appendix). The interviews lasted for 30 to 45 minutes and they were audio-recorded using a smartphone while filed notes were also taken on body language and feelings of the participant at the time of data collection.

### Data management and analysis

Data were transcribed verbatim and the transcripts were verified against the audio recordings to clarify any unclear information before conducting the next sets of interviews. Follow-up interviews were organized with respondents. Semantic data coding was independently done following the guidelines for the inductive thematic analysis described by Braun and Clarke [30]. Themes were generated based on the frequency of codes and sufficiency of data extracts to back up the codes. Discrepancies in codes and themes were resolved through group discussions involving the authors. Data were presented as direct quotes while providing a contextual understanding of HIV-related stigma and coping strategies among the study participants.

### Trustworthiness

Four strategies and principles of credibility, dependability, confirmability, and transferability were used to ensure and maintain trustworthiness in our study [31]. Credibility was ensured and maintained by performing member checks and prolonged engagements with the study participants while observing and noting body language during the engagements. Member checking was done by restating and summarizing information during the interviews. Dependability and confirmability were ensured by audio-recording of interviews and taking field notes against which findings and interpretations could later be tested. Group discussions in moments of disagreement during data analysis also ensured the dependability and confirmability of our data. Finally, transferability was maintained through identifying a sufficient sample size of study participants who could fully express themselves and making a thick description of the study setting & context, study population, and research methods.

### Ethics and approvals

The study protocol was reviewed and approved by the Gulu Research Ethics Committee (GUREC-047-20). The protocol was further cleared for data collection by the Uganda National Council for Science and Technology (RESCLEAR/01). Permission was sought from the Chief Administrative Officer & District Health Officer of Kole District, and the in-charge of EMTCT clinic, Aboke Health Center IV. Written informed consent was obtained from all the participants. Privacy and confidentiality were maintained during the entire process and the participants had the right to withdraw from the study without any notification or consequences. Standard operating procedures of coronavirus disease 2019 (COVID-19) prevention such as social distancing, hand washing, and wearing of face-masks were maintained through the study process.

## Results

### Participants’ characteristics

Although we conducted a total of 15 in-depth interviews, only 12 in-depth interviews were analyzed. The other three interviews were discarded because the audio recordings were corrupted and thus missing critical pieces of the interview. Nonetheless, the 12 interviews gave us sufficient information to warrant further analysis without going back to the field to collect more data. Table 1 describes the study participants.

**Table 1:** Description of the study participants.

Participants	Age in years	Level of education	Marital status	Parity*	Occupation	Duration with HIV in years
**P1**	24	Primary	Divorced	04	Subsistence farmer	15
**P2**	22	Secondary	Married	00	Business	01
**P3**	33	Primary	Married	05	Business	03
**P4**	25	Primary	Married	02	Subsistence farmer	02
**P5**	22	Primary	Divorced	04	Subsistence farmer	01
**P6**	25	Primary	Married	02	Business	01
**P7**	17	Primary	Single	00	Business	02
**P8**	24	Primary	Divorced	01	Subsistence farmer	01
**P9**	25	Secondary	Single	01	Subsistence farmer	14
**P10**	33	Secondary	Married	03	Business	01
**P11**	31	Primary	Married	05	Subsistence farmer	02
**P12**	35	No formal education	Married	04	Subsistence farmer	11

*Parity is the number of times a woman has given a birth to alive or dead baby who had reached viability (> 28 weeks of gestation for Uganda’s context) [32]; primary education is equivalent to grade one to seven; secondary education is equivalent to grade eight to twelve

### HIV-related enacted stigma experiences among pregnant women

We identified 18 codes under HIV-related stigma experiences among pregnant women. Overall, two themes of public ridicule and social rejection were generated as HIV-related stigma experiences among pregnant women in Kole District, northern Uganda.

#### Social rejection

Social rejection was defined as actual or attempted acts of excluding HIV-positive pregnant women from social relations and or social interactions in a manner that was noticeable by the victim. Social rejection was a big part of the negative experiences that HIV-positive pregnant women went through. The majority of women were rejected and isolated by members of their community or their family members. The social rejection experienced by these pregnant women are typified by expressions from some of these participants:

“*What annoys me is [that] sometimes people don’t want to associate with you*, *even with your children some time they don’t like [them]*. *That one annoys me the more*. *Sometimes people avoid your place”*. **Participant 3**

*“Sometimes you may go home and people refuse to eat together with you and people don’t allow you to carry their children”*. **Participant 7**

A 22-years-old first-time pregnant woman added her experience with social rejection:

“*People do not even want to take water from…*.*people keep fearing you*. *These things make me wonder whether I should be alive or dead…*..*I am getting used to it anyway*. *But these days it is not rampant like those days*”. **Participant 2**

These experiences bothered these women and they implicitly expressed their reservation of reaching out to such persons to share their problems. These experiences could probably undermine their willingness to seek medical care and social support in the future.

#### Public ridicule

We defined public ridicule as contemptuous and dismissive language and or behaviours. Appallingly, there is still overwhelming public ridicule of HIV-positive persons in rural Uganda and pregnant women are no exception. In this study, the majority of the participants expressed public ridicule as the number one form of HIV-related stigma experience. These experiences are explicated by these quotes from some of the participants:

*“People keep on abusing me that even you who can die anytime you are also talking*. *I feel like responding but maybe they are right…*.*after all my uncle also recently died of AIDS”*. **Participant 2**

*“Look at this person*, *she has just come and is still very young but she is already on medication and yet for us who are already old are still healthy*. *I wish they listened to advise like us those days”*. **Participant 6**

A similar experience was echoed by a 17-year old first-time pregnant woman who to part in the study:

*“The one which annoyed me most was a statement from people that I started taking at early age and comments from my brother that I refused to listen to their advice”*. **Participant 8**

Community members are also fond of ridiculing pregnant women that they will transmit HIV to their unborn children as elucidated by this 31-year old participant woman has now given birth five times:

*“When I go to fetch water*, *I sometimes hear people bickering that that so and so’s wife is HIV positive*. *That my husband is very unfortunate because all my children will have HIV”*. **Participant 11**

#### HIV-related stigma coping strategies among pregnant women

We identified 39 codes under HIV-related stigma coping strategies among pregnant women from the 12 interviews. Out of these codes, ignoring, social support, and prayers were generated as themes for HIV-related stigma coping strategies among pregnant women in Kole District, northern Uganda. These themes are expounded below:

#### Ignoring

Ignoring as a theme was defined as intentionally refusing to take notice of or acknowledge stigma directed to a participant. The majority of the respondents coped with HIV-related stigma experienced by simply ignoring what people said about them or did to them. This is epitomized by the statements from some of these participants:

*“*. . . .*I thought about it but later I decided to ignore what people were saying and concentrate on taking my drugs*. *My drugs gave me assurance that I can still live for long despite peoples’ words*. *I just kept on ignoring”*. **Participant 8**

When participant 6, a 25-year-old married pregnant woman with two children was probed how she coped with the enacted stigma directed towards her, she simply responded that;

*“*. . . .*For me*, *I don’t respond to such because I know it can affect anyone*, *his may even be worse than mine”*. **Participant 6**

Participant, 9 who was diagnosed with HIV 14 years ago clarified that her focus was on living a descent life instead of focusing on negative comments from people.

*“…*.*That’s how I feel because I have to be free*, *I have to live like other people without thinking of how I am*, *and that might bring me another sickness”*. **Participant 9**

Although most of the participants had learned to ignore stigma and discrimination directed towards them, most of them reported that they were initially disturbed by negative comments from family and community members before they developed some of these coping mechanisms.

#### Social support

Social support was defined as psychological, emotional, and material support provided by friends, family, and peers to help cope with stigma experiences. Respondents reported that they gained a lot of support from friends, family members, and intimate partners even after the HIV diagnosis. In particular, support from the husband and close relatives was invaluable for many respondents. One respondent who had recently tested positive for HIV described how her mother and husband has been supportive to her:

“*I always consult my mother*, *my own mother*. *I tell her what is hurting me*, *she teaches me*, *and talks to me*. *He (husband) is HIV negative*, *but he always encourages me to keep taking my drugs and forget about the bad them people are saying about me*.*”* **Participants 2**

Some women on the other hand gained invaluable support from community members who helped them deal with the fears of living positively in the face of public ridicule. This is epitomized by a quote from a 17-year-old single mother:

“*Some of the community members assisted me to keep living*. *They gave me material things including food*. *I have friends I talk to and encourage me and I feel better because they don’t see me differently*, *nothing changed anyway*. *I thank God for them every day because without them I would have given up”* **Participant 7**

Additionally, the EMTCT clinic became a very good meeting place for some of these participants to link up with their peers from where they shared their HIV- related stigma experiences and how they were coping with them. This is exemplified by a comment from one of the participants:

*“If you come for getting the drugs you find the people you know also*, *sometimes other complicated diseases kill you but not even AIDs*. *I look for a friend who is also like me and I stay with”*. **Participant 3**

#### Prayers

Prayer as a theme was conceptualized as an act of seeking divine intervention either individually or in groups for comfort in moments of distress emanating from HIV-related stigma experiences. During our interviews, it became apparent that prayer was a common way of coping with HIV-related experiences among the study participants. This was demonstrated by extracts of comments from some of these participants:

*“……*..*I also pray to God to add me more days*, *I love to pray*. *I am a catholic that is where I pray*. *I normally ask God to forgive me because it was not my intention to contract HIV*. *Of course*, *sometimes I feel down but when I talk to my God to give me strengths to overcome what people are doing to me*, *I feel much better and in control”*. **Participant 12**

Although the participants held the doctors’ advice and or consolation in high regard, most of them still felt that the divine power was the superior means of overcoming the stigma and discrimination experiences they were experiencing. This is epitomized by this quote from a 33-year old married woman who had five children:

*“……*..*the doctors have been very supportive in this facility*. *One day I came crying in his office*, *and when he asked me why I was crying*, *I told him what I was going through [stigma and discrimination]*. *He was very compassionate and supportive*. *But for me with these things [stigma and discrimination] now*, *the most important thing is prayers and following the doctors’ advice”*. **Participant 3**

## Discussion

We aimed to explore HIV-related stigma experiences and coping strategies among pregnant in rural northern Uganda. Social rejection and public ridicule were identified as HIV-related stigma experiences while ignoring, social support, and prayers were identified as HIV-related coping strategies among the study participants.

Our results showed that social rejection was a big part of the negative experiences that HIV-positive pregnant women went through. The women living with HIV are shunned by family, peers, and wider community while others face poor treatment which has mental health problems. HIV-positive women are normally held responsible for their condition and therefore social rejection is almost inevitable [33]. Such conditions limit disclosure of HIV status, access to HIV testing, treatment, and other HIV services [34]. This isolation that social rejection brings can lead to low self-esteem, depression, and suicide [35]. Our results mirror the report by the International Center for Research on Women in Bangladesh, Ethiopia, and the Dominican Republic indicating that many women living HIV have experienced social rejection [35]. Our results also echo the findings of a survey conducted in five continents showing that one-third of HIV-positive patients experienced social rejection [36]. These findings suggests that more effort is still required to eradicate social rejection of persons living with HIV.

In this study, the majority of the participants expressed public ridicule as another form of HIV-related stigma experience. There is still overwhelming public ridicule of HIV-positive persons in rural Uganda and pregnant women are no exception. HIV-positive pregnant women are increasingly stigmatized and marginalized from society. This result suggests that stigma is prevalent in the context of HIV measured in terms of the attitude of those who are not infected. This is reflected in a range of behaviours such as labeling, insulting, avoidance, and negative stereotype which is in line with previous studies [37]. A survey in 2014 in Ghana assessing respondents’ level of stigma against people living with HIV showed that only 8% and 14% of women and men respectively had positive attitudes overall to stigma indicators [38]. Also similar to our findings, a survey of married HIV-positive women in India showed that 88% of the participants experienced stigma from the community particularly the neighbors [39]. These findings suggest that a big portion of the rural population still do not view HIV/AIDS as a chronic condition with many modes of transmission but rather a lethal disease emanating from one’s promiscuity.

Our results revealed that the majority of the respondents coped with stigma and discrimination experienced by simply ignoring what people said about them or did to them. Coping by ignoring is a passive way of protecting oneself. Researchers believe that being protective in the disclosure may expose the stigmatized individuals to more stigma including violence and thus adopting a passive strategy [40, 41]. Our results mirror the findings of a study conducted in five African countries and parts of Spain on HIV-related stigma coping strategies [40, 42]. Ignoring is a negative coping strategy that may affect the psychological wellbeing of HIV-positive pregnant women [42]. Therefore, HIV positive pregnant women should be supported to identify better HIV-related stigma coping strategies.

Our findings indicated that social support was one of the positive coping strategies adopted by HIV-positive pregnant women. The respondents reported that they gained a lot of support from friends, family members, and intimate partners even after their HIV diagnosis. In particular, support from the husband and close relatives was invaluable for many respondents. Studies assessing social support and stigma among HIV-positive clients point to the role of social support as a buffering factor [43]. Consistent with our results, a meta-analysis in North America demonstrated that high levels of stigma were significantly associated with low social support and poor psychological outcome [44]. These findings are also consistent with the results of other studies conducted in Uganda [45, 46] among HIV-positive women during pregnancy and the postpartum period. According to a systematic review by Beres, Narasimhan [47], informal social support from family, friends, and relatives are by far one of the best copying strategy used by women living with HIV. Justifiably, social support has been shown to promote adherence to antiretroviral therapy which is positive for PLWHIV [48, 49]. These findings suggest that social support is invaluable to HIV-positive pregnant women in mitigating HIV-related stigma experiences.

In our findings, it became apparent that prayer was a common way of coping with HIV-related experiences among the study participants. Prayers significantly shape an individual’s outlook on living HIV-related stigma. Prayers seem to provide a sense of peace and hope and help people to accept any condition [50]. Prayers and faith in God have been cited as major coping strategies for coping with HIV-related stigma in Uganda and other African countries [40, 51]. Therefore, it is imperative that HIV-positive pregnant women are linked to spiritual support networks as a way of coping with HIV-related stigma experiences.

### Limitations of the study

This was a qualitative study are therefore the results of this study may not be generalizable to other contexts which are not identical to that of this study. Additionally, social-desirability bias could have been introduced during our in-depth interviews since the interviews were conducted within the healthcare facility thus preventing the emergence of healthcare-based HIV-related stigma and discrimination experiences.

## Conclusions

Enacted stigma in the form of social rejection and public ridicule was the dominant type of HIV-related stigma experienced by HIV-positive pregnant women in rural northern Uganda. Meanwhile, HIV-positive pregnant women coped with these challenges through ignoring, social support, and prayers. The results of this study give insight into the dominant form of HIV-related stigma among pregnant women in rural Uganda and how they cope with the problem. These results can be used to inform the design of future interventions to mitigate HIV-related stigma experiences among pregnant women. Future studies should compare the current results with research previously done studies to see if there are differences in HIV-related stigma experienced over the years as the context of HIV care continues to evolve with the advent of antiretroviral therapy. More research is also needed to elucidate the link between HIV-related stigma experiences and mother-to-child transmission of HIV among rural pregnant women.

## Supporting information

S1 Appendix In-depth interview guide. (DOCX)

## References

[1] Avert. HIV stigma and discrimination 2019 [cited 2019 March]. Available from: https://www.avert.org/professionals/hiv-social-issues/stigma-discrimination#footnote72_jnxi6za.

[2] Centers for Disease Control and Prevention. HIV stigma and discrimination 2021 [cited 2021 January]. Available from: https://www.cdc.gov/hiv/basics/hiv-stigma/index.html.

[3] Liamputtong P, Liamputtong P. Stigma, discrimination and living with HIV/AIDS: A cross-cultural perspective: Springer; 2013.

[4] UNAID Data. Global HIV & AIDS statistics—2018 fact sheet. UNAIDS org (http://www/unaids/org/en/resources/fact-sheet)[Accessed August 8 2018] Export Citation. 2018.

[5] Wirawan DN. Stigma and discrimination: Barrier for ending AIDS by 2030 and achieving the 90-90-90 targets by 2020. Public Heal Prev Med Arch. 2019.

[6] Hampson ME, Watt BD, Hicks RE. Impacts of stigma and discrimination in the workplace on people living with psychosis. BMC Psychiatry. 2020;20(1):1–11. 32513133 10.1186/s12888-020-02614-z PMC7278154

[7] Crockett KB, Kalichman SC, Kalichman MO, Cruess DG, Katner HP. Experiences of HIV-related discrimination and consequences for internalised stigma, depression and alcohol use. Psychology & health. 2019;34(7):796–810. doi: 10.1080/08870446.2019.1572143 30773914 PMC6557669

[8] Lipira L, Williams EC, Huh D, Kemp CG, Nevin PE, Greene P, et al. HIV-related stigma and viral suppression among African-American women: exploring the mediating roles of depression and ART nonadherence. AIDS and Behavior. 2019;23(8):2025–36. doi: 10.1007/s10461-018-2301-4 30343422 PMC6815932

[9] Chikhungu L, Bispo S, Newell M. Postnatal HIV transmission rates at age six and 12 months in infants of HIV-infected women on ART initiating breastfeeding: a systematic review of the literature. Commissioned for the guideline review. Guideline: updates on HIV and infant feeding: the duration of breastfeeding, and support from health services to improve feeding practices among mothers living with HIV Edn Geneva: World Health Organization. 2016. 27583316

[10] Grossman CI, Stangl AL. Global action to reduce HIV stigma and discrimination. Wiley Online Library; 2013. p. 18881. 10.7448/IAS.16.3.18881 PMC3833103 24242269

[11] Nyblade L, Stangl A, Weiss E, Ashburn K. Combating HIV stigma in health care settings: what works? Journal of the international AIDS Society. 2009;12(1):1–7. doi: 10.1186/1758-2652-12-15 19660113 PMC2731724

[12] Stangl AL, Lloyd JK, Brady LM, Holland CE, Baral S. A systematic review of interventions to reduce HIV‐related stigma and discrimination from 2002 to 2013: how far have we come? Journal of the International AIDS Society. 2013;16:18734. doi: 10.7448/IAS.16.3.18734 24242268 PMC3833106

[13] Feyissa GT, Abebe L, Girma E, Woldie M. Stigma and discrimination against people living with HIV by healthcare providers, Southwest Ethiopia. BMC Public Health. 2012;12(1):1–12. doi: 10.1186/1471-2458-12-522 22794201 PMC3506482

[14] Zarei N, Joulaei H, Darabi E, Fararouei M. Stigmatized attitude of healthcare providers: a barrier for delivering health services to HIV positive patients. International journal of community based nursing and midwifery. 2015;3(4):292. 26448956 PMC4591575

[15] Stringer KL, Turan B, McCormick L, Durojaiye M, Nyblade L, Kempf M-C, et al. HIV-related stigma among healthcare providers in the deep south. AIDS and Behavior. 2016;20(1):115–25. doi: 10.1007/s10461-015-1256-y 26650383 PMC4718797

[16] Bond V, Chase E, Aggleton P. Stigma, HIV/AIDS and prevention of mother-to-child transmission in Zambia. Evaluation and program planning. 2002;25(4):347–56.

[17] Parsons JA, Bond VA, Nixon SA. ‘Are we not human?’Stories of stigma, disability and HIV from Lusaka, Zambia and their implications for access to health services. PloS one. 2015;10(6):e0127392. doi: 10.1371/journal.pone.0127392 26039666 PMC4454491

[18] Hargreaves JR, Krishnaratne S, Mathema H, Lilleston PS, Sievwright K, Mandla N, et al. Individual and community-level risk factors for HIV stigma in 21 Zambian and South African communities: analysis of data from the HPTN071 (PopART) study. AIDS (London, England). 2018;32(6):783. doi: 10.1097/QAD.0000000000001757 29369164 PMC5854764

[19] Krishnaratne S, Bond V, Stangl A, Pliakas T, Mathema H, Lilleston P, et al. Stigma and judgment toward people living with HIV and key population groups among three cadres of health workers in South Africa and Zambia: analysis of data from the HPTN 071 (PopART) trial. AIDS patient care and STDs. 2020;34(1):38–50. doi: 10.1089/apc.2019.0131 31944852 PMC6983735

[20] Chan BT, Weiser SD, Boum Y, Siedner MJ, Mocello AR, Haberer JE, et al. Persistent HIV-related stigma in rural Uganda during a period of increasing HIV incidence despite treatment expansion. AIDS (London, England). 2015;29(1):83. doi: 10.1097/QAD.0000000000000495 25268886 PMC4286463

[21] Tsai AC, Bangsberg DR, Kegeles SM, Katz IT, Haberer JE, Muzoora C, et al. Internalized stigma, social distance, and disclosure of HIV seropositivity in rural Uganda. Annals of Behavioral Medicine. 2013;46(3):285–94. doi: 10.1007/s12160-013-9514-6 23690283 PMC3795853

[22] Kuteesa MO, Wright S, Seeley J, Mugisha J, Kinyanda E, Kakembo F, et al. Experiences of HIV-related stigma among HIV-positive older persons in Uganda–a mixed methods analysis. SAHARA-J: Journal of Social Aspects of HIV/AIDS. 2014;11(1):126–37. doi: 10.1080/17290376.2014.938103 25053275 PMC4272102

[23] Kalichman SC, Shkembi B, Wanyenze RK, Naigino R, Bateganya MH, Menzies NA, et al. Perceived HIV stigma and HIV testing among men and women in rural Uganda: a population-based study. The Lancet HIV. 2020;7(12):e817–e24. doi: 10.1016/S2352-3018(20)30198-3 32910903 PMC9706443

[24] Nyblade L, Stockton MA, Giger K, Bond V, Ekstrand ML, Mc Lean R, et al. Stigma in health facilities: why it matters and how we can change it. BMC medicine. 2019;17(1):1–15. 30764806 10.1186/s12916-019-1256-2 PMC6376713

[25] Doyle L, McCabe C, Keogh B, Brady A, McCann M. An overview of the qualitative descriptive design within nursing research. Journal of Research in Nursing. 2020;25(5):443–55. doi: 10.1177/1744987119880234 34394658 PMC7932381

[26] Holly C. Scholarly inquiry and the DNP capstone: Springer Publishing Company; 2013.

[27] Ritchie J, Lewis J, Elam RG. Selecting samples. Qualitative research practice: A guide for social science students and researchers. 2013;111.

[28] Malterud K, Siersma VD, Guassora AD. Sample size in qualitative interview studies: guided by information power. Qualitative health research. 2016;26(13):1753–60. doi: 10.1177/1049732315617444 26613970

[29] Turan J, Nyblade L, Monfiston P. Stigma and Discrimination: Key Barriers to Achieving Global Goals for Maternal Health and the Elimination of New Child HIV Infections. Working Paper, 2012.

[30] Braun V, Clarke V. Using thematic analysis in psychology. Qualitative research in psychology. 2006;3(2):77–101.

[31] Denzin NK, Lincoln YS. The Sage handbook of qualitative research (5th edition): sage; 2018.

[32] Ministry of Health. Uganda Clinical Guidance 2016: National Guidelines for Management of Common Conditions. 2016.

[33] Cook RJ, Dickens BM. Reducing stigma in reproductive health. International Journal of Gynecology & Obstetrics. 2014;125(1):89–92. doi: 10.1016/j.ijgo.2014.01.002 24513258

[34] Katz IT, Ryu AE, Onuegbu AG, Psaros C, Weiser SD, Bangsberg DR, et al. Impact of HIV‐related stigma on treatment adherence: systematic review and meta‐synthesis. Journal of the International AIDS Society. 2013;16:18640. doi: 10.7448/IAS.16.3.18640 24242258 PMC3833107

[35] The Well Project. Stigma and Discrimination Against Women Living with HIV 2016 [cited 2022 30 January]. Available from: https://www.thewellproject.org/hiv-information/stigma-and-discrimination-against-women-living-hiv.

[36] Nachega JB, Morroni C, Zuniga JM, Sherer R, Beyrer C, Solomon S, et al. HIV-related stigma, isolation, discrimination, and serostatus disclosure: a global survey of 2035 HIV-infected adults. Journal of the International Association of Physicians in AIDS Care. 2012;11(3):172–8. doi: 10.1177/1545109712436723 22431893

[37] Mahamboro DB, Fauk NK, Ward PR, Merry MS, Siri TA, Mwanri L. HIV stigma and moral judgement: qualitative exploration of the experiences of HIV stigma and discrimination among married men living with HIV in Yogyakarta. International journal of environmental research and public health. 2020;17(2):636. doi: 10.3390/ijerph17020636 31963807 PMC7013688

[38] Ghana Statistical Service GHS, ICF International. Ghana demographic and health survey 2014. Rockville, Maryland, USA: GSS, GHS, and ICF International. 2015.

[39] Halli SS, Khan CH, Moses S, Blanchard J, Washington R, Shah I, et al. Family and community level stigma and discrimination among women living with HIV/AIDS in a high HIV prevalence district of India. Journal of HIV/AIDS & Social Services. 2017;16(1):4–19.

[40] Makoae LN, Greeff M, Phetlhu RD, Uys LR, Naidoo JR, Kohi TW, et al. Coping with HIV-related stigma in five African countries. Journal of the Association of Nurses in AIDS Care. 2008;19(2):137–46. doi: 10.1016/j.jana.2007.11.004 18328964 PMC2346777

[41] Varni SE, Miller CT, McCuin T, Solomon S. Disengagement and engagement coping with HIV/AIDS stigma and psychological well-being of people with HIV/AIDS. Journal of social and clinical psychology. 2012;31(2):123–50. doi: 10.1521/jscp.2012.31.2.123 22611302 PMC3355931

[42] Sanjuán P, Molero F, Fuster MJ, Nouvilas E. Coping with HIV related stigma and well-being. Journal of Happiness Studies. 2013;14(2):709–22.

[43] Rao D, Chen W-T, Pearson CR, Simoni JM, Fredriksen-Goldsen K, Nelson K, et al. Social support mediates the relationship between HIV stigma and depression/quality of life among people living with HIV in Beijing, China. International journal of STD & AIDS. 2012;23(7):481–4. doi: 10.1258/ijsa.2009.009428 22844001 PMC3408622

[44] Logie C, Gadalla T. Meta-analysis of health and demographic correlates of stigma towards people living with HIV. AIDS care. 2009;21(6):742–53. doi: 10.1080/09540120802511877 19806490

[45] Mutumba M, Bauermeister JA, Harper GW, Musiime V, Lepkowski J, Resnicow K, et al. Psychological distress among Ugandan adolescents living with HIV: Examining stressors and the buffering role of general and religious coping strategies. Global public health. 2017;12(12):1479–91. doi: 10.1080/17441692.2016.1170871 28278753

[46] Ashaba S, Kaida A, Burns BF, O’Neil K, Dunkley E, Psaros C, et al. Understanding coping strategies during pregnancy and the postpartum period: a qualitative study of women living with HIV in rural Uganda. BMC pregnancy and childbirth. 2017;17(1):1–10. 28482821 10.1186/s12884-017-1321-9 PMC5423027

[47] Beres LK, Narasimhan M, Robinson J, Welbourn A, Kennedy CE. Non-specialist psychosocial support interventions for women living with HIV: A systematic review. AIDS care. 2017;29(9):1079–87. doi: 10.1080/09540121.2017.1317324 28438030 PMC6957075

[48] Atukunda EC, Musiimenta A, Musinguzi N, Wyatt MA, Ashaba J, Ware NC, et al. Understanding patterns of social support and their relationship to an ART adherence intervention among adults in rural Southwestern Uganda. AIDS and Behavior. 2017;21(2):428–40. doi: 10.1007/s10461-016-1559-7 27671479 PMC5288444

[49] Mao Y, Qiao S, Li X, Zhao Q, Zhou Y, Shen Z. Depression, social support, and adherence to antiretroviral therapy among people living with HIV in Guangxi, China: A longitudinal study. AIDS Education and Prevention. 2019;31(1):38–50. doi: 10.1521/aeap.2019.31.1.38 30742482

[50] Genrich GL, Brathwaite BA. Response of religious groups to HIV/AIDS as a sexually transmitted infection in Trinidad. BMC Public Health. 2005;5(1):1–12. doi: 10.1186/1471-2458-5-121 16288659 PMC1310629

[51] Mutumba M, Bauermeister JA, Musiime V, Byaruhanga J, Francis K, Snow RC, et al. Psychosocial challenges and strategies for coping with HIV among adolescents in Uganda: a qualitative study. AIDS patient care and STDs. 2015;29(2):86–94. doi: 10.1089/apc.2014.0222 25607900

