# Supplementary material for: HIV-related stigma experiences and coping strategies among pregnant women in rural Uganda: A qualitative descriptive study
Source: PLoS One. 2022 Oct 7;17(10):e0272931. doi: 10.1371/journal.pone.0272931 (PMC9543605; doi:10.1371/journal.pone.0272931)
Supplement: S1 Appendix — (DOCX) [file pone.0272931.s001.docx]

| **Title** | HIV-related stigma experiences and coping strategies among pregnant women in rural Uganda: a qualitative descriptive study |
| --- | --- |
| **Respondent ID** |  |
| **Date of the interview** |  |
| **Socio-demographic characteristics**  **(Retrieved from survey results)** | 1. Age 2. Level of education 3. Marital status 4. Number of children 5. Occupation 6. Duration with HIV |
| **Probes** | **Specific probes will depend on how the interview is unfiolding** |
| **HIV-related stigma experiences** | 1. What is the experience like as a pregnant woman who is HIV positive? |
| **Coping strategies** | 1. How do you handle these negative experiences? |
| **Closure** | I think we are coming to the end of this conversation, is there anything more you would like to add?  You shared A, B, and C regarding your experiences as a pregnant woman who is HIV positive and how you cope with some of these negative experiences. Is this a fair representation of what we talked about?  Thank you for your time and participation. |
